# Structural integrity and viability of *Fredericella sultana* statoblasts infected with *Tetracapsuloides bryosalmonae* (Myxozoa) under diverse treatment conditions

**DOI:** 10.1186/s13567-017-0427-4

**Published:** 2017-04-05

**Authors:** Ahmed Abd-Elfattah, Mansour El-Matbouli, Gokhlesh Kumar

**Affiliations:** 1 Clinical Division of Fish Medicine, University of Veterinary Medicine, Vienna, Austria; 2 Institute of Parasitology, University of Veterinary Medicine, Vienna, Austria

## Abstract

*Fredericella sultana* is an invertebrate host of *Tetracapsuloides bryosalmonae*, the causative agent of proliferative kidney disease in salmonids. The bryozoan produces seed-like statoblasts to facilitate its persistence during unfavourable conditions. Statoblasts from infected bryozoans can harbor *T. bryosalmonae* and give rise to infected bryozoan colonies when conditions improve. We aimed in the present study to evaluate the integrity and viability of *T. bryosalmonae*-infected statoblasts after a range of harsh treatment conditions. We tested if statoblasts could survive ingestion by either brown trout or common carp. After ingestion, the fish faeces was collected at different time points. We also tested physical stressors: statoblasts collected from infected colonies were desiccated at room temperature, or frozen with and without Bryozoan Medium C (BMC). After treatments, statoblasts were assessed for physical integrity before being incubated on BMC to allow them to hatch. After 4 weeks, hatched and unhatched statoblasts were tested by PCR for the presence of the parasite. We found that statoblasts ingested by brown trout and those frozen in BMC were completely broken. In contrast, statoblasts ingested by common carp and those subjected to dry freezing were able to survive and hatch. *T. bryosalmonae* was detected by PCR in both hatched and unhatched infected statoblasts, but neither from broken nor uninfected statoblasts. Our results confirmed for the first time the ability of infected statoblasts to survive passage through a fish, and freezing. These findings suggest potential pathways for both persistence and spread of *T. bryosalmonae*-infected statoblasts in natural aquatic systems.

## Introduction

Bryozoans are sessile, aquatic, colonial invertebrates, which are found in many fresh water and marine environments [1]. Freshwater species (Class Phylactolaemata) are common in lakes and rivers, and are found attached to submerged tree branches, roots, rocks etc. [2, 3]. Although some species reproduce sexually, by producing swimming larvae, more commonly bryozoans propagate asexually via colony fragmentation, budding and by producing dormant stages called statoblasts. These hard, seed-like structures are typically 1–2 mm across, are produced in large numbers and have resistant chitin valves [4]. Statoblasts can survive a range of unfavorable environmental stressors [3, 5], then when conditions improve, they can germinate to form a new individual zooid. Statoblasts can be distributed passively within and between water bodies by movement of waterfowl and fish [5].

Statoblasts are responsible for vertical transmission of the myxozoan parasite, *Tetracapsuloides bryosalmonae*, the causative agent of proliferative kidney disease (PKD) in salmonid fish [6]. The parasite can be transmitted through bryozoan colony fragmentation, infected statoblasts and infected brown trout, *Salmo trutta* [3, 6, 7]. Although some salmonid and cyprinid species were tested positive per PCR for *T. bryosalmonae*, their role in the dispersal of the parasite is still yet unknown since their spore releasing ability is still under investigation (Authors own unpublished data). *Fredericella sultana* is the most studied bryozoan species known to host the parasite. This bryozoan is found at a wide range of temperatures and can tolerate cold environments [8, 9], with *F. sultana* statoblasts shown to have some resistance to both freezing and desiccation [10].

Bryozoan statoblasts have been found in the intestine of waterfowl [11–14] and fecal material of some fish species [15]. Statoblasts were found to hatch after passing through the digestive tract of pintail (*Anas acuta*), shoveler (*Anas clypeata*) and mallard (*Anas platyrhynchos*), which suggests endozoochorous dispersal of viable statoblast is possible [11]. Scherbak and Karaeva [16] found that bryozoan statoblasts of two species (*Plumatella fungosa* and *P. repens*) could remain viable after passage through the intestinal tract of common carp (*Cyprinus carpio*), a species capable of long distance movement [17]. The role of common carp and brown trout in the dispersal of statoblasts infected with *T. bryosalmonae* has not been investigated.

To date, the viability of statoblasts infected with *T. bryosalmonae* under harsh environmental conditions has not been investigated. We hypothesize that the ability of infected statoblasts to survive desiccation and freezing, and be distributed through wide-ranging hosts, could explain both the wide spread distribution of the parasite and the presence of infected bryozoan populations at sites free of salmonids [3, 18]. Therefore, the aims of the present study were to compare the viability of statoblasts collected from both uninfected *F. sultana* colonies and those infected with *T. bryosalmonae*, after exposure to physical (freezing, desiccation) and biological (ingestion by two fish species) stressors.

## Materials and methods

### Ethics statement

This study was approved by the institutional ethics committee of the University of Veterinary Medicine Vienna, and the national authority according to §26 of the Austrian Law for Animal Experiments, Tierversuchsgesetz 2012—TVG 2012 91 under the No. GZ 68.205/0247-II/3b/2011.

### Collection of *F. sultana* statoblasts

**Figure 1:**
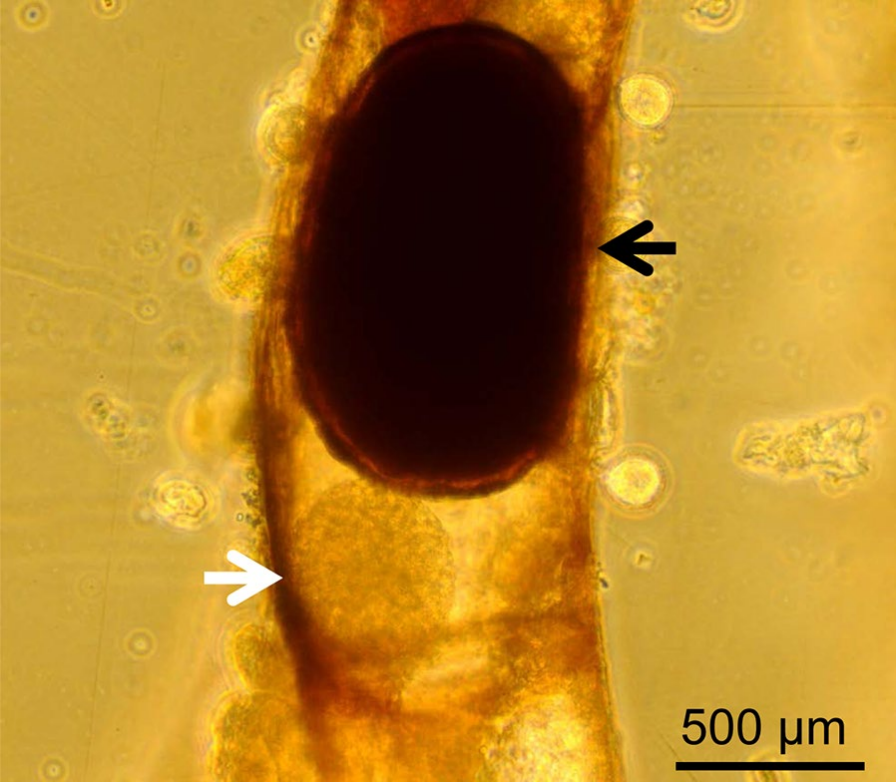
**Association of statoblast with a mature sac of** ***Tetracapsuloides bryosalmonae.*** Zooid from an infected *Fredericella sultana* colony showing a statoblast (black arrow) in close association with a mature sac of *Tetracapsuloides bryosalmonae* (white arrow).

Specific pathogen free (SPF) *F. sultana* colonies were raised from statoblasts obtained from our established laboratory cultures, which originated at the very beginning from a single largely clonal population in Germany [19]. We cohabitated these colonies with *T. bryosalmonae*-infected brown trout (*N* = 10) for 3 weeks, in several batches, and maintained them under optimal laboratory conditions [19]. Colonies were examined before, during and after the cohabitation using a dissecting microscope (Olympus SZ-PT) to check for the presence of overt infections: sac-like stages in the zooids and covert infections that consist of swirling single-cell *T. bryosalmonae* stages [3]. Prior to the start of the experiments, six zooids each were collected from an uninfected and an infected colony, then tested by nested PCR to verify infection status [20, 21]. Infected colonies had both covert and overt (Figure 1) infections in zooids. Statoblasts were collected from these infected and also SPF uninfected colonies; these statoblasts are henceforth referred to as “infected” and “uninfected” statoblasts. The two groups of statoblasts were kept separately in Petri dish plates filled with Bryozoan Medium C (BMC) [19] at 4 °C until further use.

### Viability of statoblasts after passage through fish

Brown trout and common carp were used to investigate their potential roles in dispersing statoblasts of *F. sultana*. Twelve brown trout (length 13 ± 1 cm) and 12 common carp (10 ± 1 cm) were used. Fish were kept separately in 100 L glass aquaria with de-chlorinated flow-through water. All fish were starved for 24 h before the feeding experiments. Infected and uninfected statoblasts were mixed separately with 4 pellets (4 mm) of commercial fish feed and this mix was injected directly into the oesophagus by gastric intubation at a dose of 15 statoblasts per fish. Six fish of each species received separately infected statoblasts and 6 received uninfected statoblasts. Fish were kept in different aquaria and checked for any abnormal behavior for 2 h. Fish were fed with the equivalent of 1% of their body weight of commercial pellet feed 6 h post-intubation (pi).

**Figure 2:**
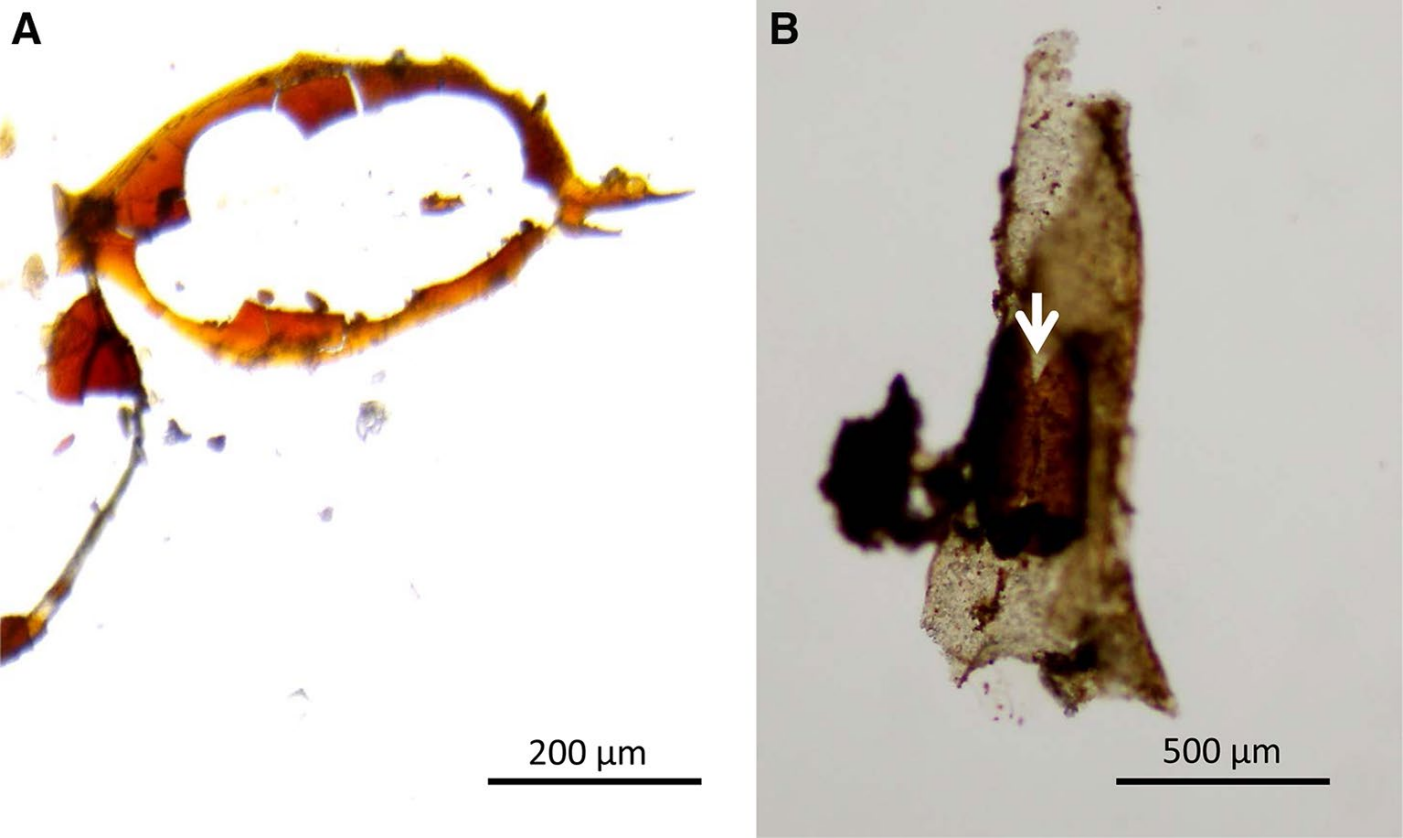
***Fredericella sultana*** **statoblasts extracted from the faeces of brown trout.** **A** Fragmented statoblast with a remnant of its outer membrane. **B** Opened statoblast inside a zooid (white arrow).

Fish faeces were collected separately from each group every 2 h. Faeces were examined under a stereomicroscope for the presence of statoblasts, which were then immediately cleaned with BMC, and counted to determine the ratio of broken (opened chitin valves, Figure 2) to intact statoblasts (unopened dark brown chitin valves). Intact statoblasts were placed in Petri dish plates filled with BMC and stored at 4 °C in a refrigerator until use for hatching (see below). Faeces sampling was terminated when no statoblasts were observed from any fish after three consecutive collections (~48 h pi).

### Desiccation and freezing of statoblasts

#### Statoblasts were tested under three treatment regimens

- Treatment 1—desiccation: triplicate groups of 30 infected and 30 uninfected statoblasts were covered in a few drops of BMC (which dried out rapidly) and kept in separate Petri dish plates, then incubated at 18 ± 1 °C for 1 week.
- Treatment 2—freezing: triplicate groups of 30 infected and 30 uninfected statoblasts were placed with or without BMC in Petri dish plates and immediately frozen at −5 ± 1 °C for 2 weeks.
- Treatment 3: to compare the viability and the hatchability rate, triplicate groups of 30 infected statoblasts (positive control) and 30 uninfected statoblasts (negative control) without any treatments were placed into Petri dish plates filled with BMC, and held at 4 °C for 2 weeks.

### Assessment of statoblast integrity, and viability through hatching

Statoblasts were first assessed for structural integrity: those with intact chitin valves as seen under a stereomicroscope were considered as intact, and were then used for viability (hatching) tests. For the desiccation and freezing treatments, BMC was added to the Petri dish plates and incubated for 24 h at 18 °C before calculating the ratio of intact and broken statoblasts. All groups of statoblasts were held at 4 °C for 2 weeks before being assessed for hatching.

To allow statoblasts to hatch, plates were transferred to aerated 10 L aquaria filled with BMC, and held at 18 °C. Once the statoblasts hatched, they were fed with a mixture of five algae species, which consisted of 80% *Cryptomonas ovata* and 20% other species (*Chlamydomonas reinhardtii*, *Synechococcus rubescens*, *Synechococcus* spp. and *Synechococcus leopoliensis*) [22]. Four weeks later, all statoblasts (hatched and unhatched) were collected to test for the presence of *T. bryosalmonae* by PCR.

### PCR

Genomic DNA was extracted from newly hatched zooids (~4 weeks old, comprising at least 2 zooids), unhatched statoblasts, and broken statoblasts from all groups, using QIAamp DNA Mini Kit (QIAGEN) according to the manufacturer’s instructions. Primers used for the detection of *T. bryosalmonae* were the following: 5F (5′-CCTATTCAATTGAGTAGGAGA-3′) and 6R (5′-GGACCTTACTCGTTTCCGACC-3′) [20] for the first round, followed by a second round PCR using PKD-real F (5′-TGTCGATTGGACACTGCATG-3′) and PKD-real R (5′-ACGTCCGCAAACTTACAGCT-3′) [21]. PCR assays were performed in triplicate. PCR amplifications were carried out in 25 μL reaction volumes containing 12.5 μL of 2× ReddyMix PCR Master Mix (ABGene), 10 pmol of each primer, 1 μL of test DNA template and rest of PCR grade water. The cycling program was: initial denaturation at 95 °C for 5 min, followed by 35 cycles of 95 °C for 1 min, 55 °C in the first round and 61 °C in the nested for 1 min, 72 °C for 1 min and a final extension step at 72 °C for 5 min. Amplicons were analyzed by electrophoresis on 1.5% agarose gels in Tris acetate–EDTA buffer stained with ethidium bromide.

### Statistical analysis

Chi Square tests were used to analyze differences between different treatments on the viability and hatching of statoblasts, including positive and negative controls. A *p* value <0.05 was considered significant. Statistical analyses were conducted using SPSS software v20.

## Results

Table 1 shows the results of statoblast integrity and viability for each treatment.

**Table 1:** **Numbers of intact and viable statoblasts under different experimental conditions**

Group	Number of intact statoblasts after treatment	Number of viable (hatched) statoblasts	PCR results
Uninfected statoblasts (negative control) without any treatments	90	60	–
Infected statoblasts (positive control) without any treatments	90	24	+
Freeze dried uninfected statoblasts without BMC	90	15	–
Freeze dried infected statoblasts without BMC	90	9	+
Freezed dried uninfected statoblasts with BMC	0	0	–
Freezed dried infected statoblasts with BMC	0	0	–
Dehydrated uninfected statoblasts	42	12	–
Dehydrated infected statoblasts	27	9	+
Ingested common carp uninfected statoblasts	63	24	–
Ingested common carp infected statoblasts	54	18	+
Ingested brown trout uninfected statoblasts	0	0	–
Ingested brown trout infected statoblasts	0	0	–

Ninety statoblasts were tested in each group.

−: PCR negative; +: PCR positive; BMC: Byrozoan Medium C.

### Effect of fish ingestion on statoblasts

**Figure 3:**
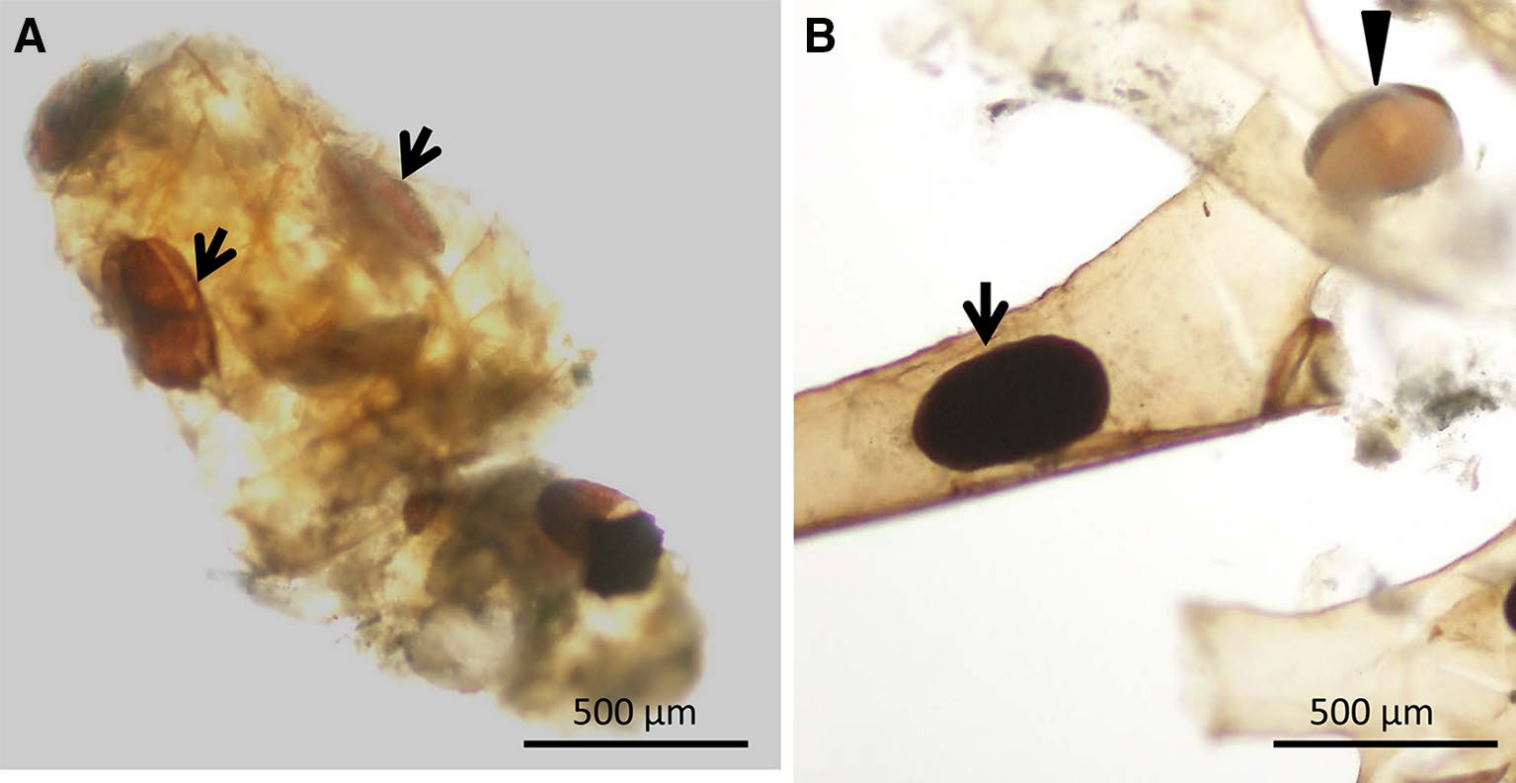
***Fredericella sultana*** **statoblast collected from the faeces of common carp.** **A** Broken statoblast (light brown, arrows) surrounded by fish intestinal mucous. **B** Intact statoblast inside a zooid (dark brown, arrow), broken statoblast with opened valve (light brown, arrowhead).

All statoblasts ingested by brown trout were found to be completely broken (Figure 2). In contrast, statoblasts ingested by common carp were either intact (dark brown) or broken (light brown with opened chitin valve) (Figure 3).

### Statoblast integrity and viability

All uninfected and infected statoblasts from the controls and those frozen without BMC remained intact. However, some of the dehydrated statoblasts were found broken. All statoblasts frozen with BMC were broken.

Viability (hatching) rates of negative control (uninfected) statoblasts was significantly (*p* < 0.000001) higher than the positive control (infected) statoblasts. The percentages of hatched negative control and positive control statoblasts were 66.66% (*N* = 60) and 26.66% (*N* = 24), respectively. Dehydrated, uninfected statoblasts were more viable (*N* = 42) than dehydrated infected statoblasts (*N* = 27) but this difference was not significant (*p* = 0.67). Statoblasts ingested by brown trout, and statoblasts frozen in BMC were excluded from the statistical analysis because they were all broken.

For the other treatment groups (frozen without BMC, dehydrated, and carp ingested), the hatching rate was slightly higher in uninfected than infected statoblasts (Table 1), but these differences were not significant; χ^2^ = 1.73, DF = 1, and *p* = 0.18, χ^2^ = 0.18 and *p* = 0.67, χ^2^ = 0.29 and *p* = 0.59, respectively.

### PCR

DNA samples from zooids (*N* = 6) collected from the uninfected colonies prior to the experiment were negative for *T. bryosalmonae*, whereas *T. bryosalmonae* was successfully amplified from zooids (*N* = 6) from infected *F. sultana*. From the treatment groups, *T. bryosalmonae* was successfully amplified from hatched infected statoblasts that had been frozen without BMC (*N* = 9), dehydrated (*N* = 9), common carp ingested (*N* = 18) and the positive control (*N* = 24). PCR detected the parasite also in unhatched infected statoblasts, which had been frozen without BMC, dehydrated, ingested by common carp, and the positive control. All broken “infected” statoblasts were negative by PCR.

## Discussion

The ability of statoblasts to survive harsh environmental conditions and subsequently hatch has been demonstrated for some bryozoan species in the class Phylactolaemata [23, 24]. To date, the effect of infection with the myxozoan parasite *T. bryosalmonae* on viability of *F. sultana* statoblasts subjected to stressful environmental conditions has not been investigated. We hypothesized that if infected statoblasts could survive harsh conditions, then this resilience could enhance dispersal and persistence of *T. bryosalmonae* in natural aquatic systems. In the present study, we compared the viability of *F. sultana* statoblasts that had been collected from either infected or uninfected bryozoan colonies, then subjected to different treatment regimens: dehydration, wet or dry freezing, and after ingestion by brown trout and common carp.

Baseline viability (hatching incidence) of untreated statoblasts from uninfected colonies was higher than statoblasts from infected colonies under laboratory conditions. This finding is in accordance with the results of Hartikainen et al. [25] but in contrast with results from a second study that showed hatching success was slightly higher in infected statoblasts compared to uninfected statoblasts from the Rivers Dun and Avon [6]. The difference could be due to yet unknown environmental factors affecting the hatchability of statoblasts. However, in the present study, statoblasts were originated from our established infected laboratory bryozoan cultures.

We determined that all hatched statoblasts from infected colonies were positive for *T. bryosalmonae* DNA by nested PCR, which suggests that there was no PCR inhibition by the statoblast chitin valves. The higher level of infection detected by nested PCR in hatched statoblasts in this study could be explained on the basis of the higher susceptibility of our laboratory maintained *F. sultana* colonies.

The amplification of *T. bryosalmonae* in 4-week old zooids hatched from infected statoblasts was in concordance with our former results [6]. Vertical transmission of the parasite through the statoblast phase of the host life cycle conveys a level of physical protection to the parasite and promotes the spread of infection to future host generations. The statoblasts resistant chitin valves act as a shield, to protect both host and parasite from not only physical stressors (freezing, drying) but also passage through the digestive tracts of some fish species [16, 26, 27].

We demonstrated that *F. sultana* statoblasts could survive desiccation, which has been shown in other bryozoans, like *Cristatella mucedo* [28]. Smyth and Reynolds [29] found that the spinoblasts (buoyant statoblasts) of *C. mucedo* and non-spinous statoblasts of *P. repens* could survive dry and other unfavourable conditions; although the incidence of hatching in their study was lower (7–10%) in contrast to our current results in dehydrated samples (28–33%). Desiccation resistance has been reported from different kinds of organisms, including nematodes, extremophile embryonic cysts, and yeasts, which enter a “cryptobiotic state” [30–33]. Different biochemical strategies are probably required in these different taxa, and in bryozoans, the non-reducing disaccharide trehalose has been linked with desiccation tolerance in statoblasts [28]. The “vitrification hypothesis” states that trehalose protects proteins and membranes, and thus enables the survival of statoblasts during environmentally stressful conditions [28].

We also demonstrated the ability of *F. sultana* statoblasts to survive freezing, which is in accordance with observations of Oda [34] who found that the statoblasts of *C. mucedo* were able to hatch after exposure to freezing temperatures. Danks et al. [35] and Duman [36] found that cold-tolerating organisms depend on their ability to use either cryoprotectants or antifreeze proteins, which lower the temperature at which water crystallizes within the organisms [37]. Hengherr and Schill [28] found in bryozoans that internal ice crystals form between −2  and −10 °C, and the amount of crystallized water in the statoblasts was about 75–80%. In our study, dry frozen statoblasts were able to hatch after exposure to −5 °C, which is in the range reported by Hengherr and Schill [28]. In contrast, statoblasts frozen wet in BMC at −5 °C were all completely broken. We propose that this occurred as the result of physical pressures imparted by crystallization of the surrounding liquid on the statoblasts.

We observed a higher hatching incidence of statoblasts after ingestion by the common carp, than after any other treatment (Table 1). The fact that common carp are herbivores (with no gastric secretions) [38], in contrast to brown trout might explain the higher viability of the statoblasts ingested by common carp. Similarly, Figuerola et al. [39] found that waterfowl with lighter gizzards and longer ceca are ideal candidates for passage of statoblasts, since the lighter gizzard is likely to destroy a fewer statoblasts before they reach the ceca. Our results were in concordance with those of Scherbak and Karaeva [16], who demonstrated that statoblasts of *P. fungosa* and *P. repens* ingested by common carp and goldfish, *Carassius carassius* were able to hatch after retrieval from the fish intestine. Additionally, our results suggest that infected statoblasts ingested by common carp can maintain the parasite infection after passage through the fish and thus demonstrate the potential role of common carp as a vector for introducing infected statoblasts to new localities. The role of animal vectors in dispersal of bryozoans is suggested by Freeland et al. [40], who found bryozoans of identical multilocus genotypes (i.e. presumed to originate from the same source population) in habitats that were some 3000 km apart. These observations, combined with our data for survival of infected statoblasts, suggest that myxozoan infections may be introduced in the same locations as where the initial colony was located or dispersed within new habitats via statoblasts of their bryozoan hosts, when transported passively by animal carriers. These might also explain the Okamura et al. [18] findings of *T. bryosalmonae*-infected bryozoan populations in places that lack salmonids.

Although many salmonid species are affected by *T. bryosalmonae*, so far only brown trout and brook trout have been shown to complete the life cycle [41–43]. The laboratory infection experiment showed that common carp are host for other myxozoans (malacosporeans) like *Buddenbrockia* spp. [44]. Field monitoring of common carp in the Czech Republic and Hungary showed a low number of *Tetracapsuloides* spp. stages and high number of *Buddenbrockia* spp. stages in different organs along with other myxozoan parasites, *Sphaerospora dykovae* and *Sphaerospora molnari* [45]. Common carp are highly mobile species, competent migrating up and down the rivers throughout the year [17]. It is capable of small and long distance movement and moved a maximum 890 km [46].

Our study provides the first evidence that infected statoblasts can remain viable under harsh conditions, which could occur in nature (freezing, desiccation and ingestion by fish). We demonstrate that infected statoblasts can survive passage through the gut of the common carp, which suggests that straying carp could transport statoblasts from one place to another in natural watersheds and thereby facilitate the spread of *T. bryosalmonae*. Also, transmission of *T. bryosalmonae* could be dispersed by infected brown trout, which release viable spores into the water bodies for more than 2 years [22] to infect bryozoan colonies. Therefore, long-distance distribution of *T. bryosalmonae* may be a natural consequence of the ability of infected host statoblasts to tolerate diverse environmental conditions.

## Abbreviations

BMC: Bryozoan Medium C
*C. mucedo*: *Cristatella mucedo*
*F. sultana*: *Fredericella sultana*
*P. repens*: *Plumatella repens*
PKD: proliferative kidney disease
*T. bryosalmonae*: *Tetracapsuloides bryosalmonae*
